# Mapping the artistic brain: Common and distinct neural activations associated with musical, drawing, and literary creativity

**DOI:** 10.1002/hbm.25025

**Published:** 2020-05-30

**Authors:** Qunlin Chen, Roger E. Beaty, Jiang Qiu

**Affiliations:** ^1^ School of Psychology Southwest University Chongqing China; ^2^ Key Laboratory of Cognition and Personality, Ministry of Education Chongqing China; ^3^ Department of Psychology Pennsylvania State University University Park Pennsylvania USA

**Keywords:** activation likelihood estimation, artistic creativity, literary creativity, musical improvisation, visual art

## Abstract

Whether creativity is a domain‐general or domain‐specific ability has been a topic of intense speculation. Although previous studies have examined domain‐specific mechanisms of creative performance, little is known about commonalities and distinctions in neural correlates across different domains. We applied activation likelihood estimation (ALE) meta‐analysis to identify the brain activation of domain‐mechanisms by synthesizing functional neuroimaging studies across three forms of artistic creativity: music improvisation, drawing, and literary creativity. ALE meta‐analysis yielded a domain‐general pattern across three artistic forms, with overlapping clusters in the presupplementary motor area (pre‐SMA), left dorsolateral prefrontal cortex, and right inferior frontal gyrus (IFG). Regarding domain‐specificity, musical creativity was associated with recruitment of the SMA‐proper, bilateral IFG, left precentral gyrus, and left middle frontal gyrus (MFG) compared to the other two artistic forms; drawing creativity recruited the left fusiform gyrus, left precuneus, right parahippocampal gyrus, and right MFG compared to musical creativity; and literary creativity recruited the left angular gyrus and right lingual gyrus compared to musical creativity. Contrasting drawing and literary creativity revealed no significant differences in neural activation, suggesting that these domains may rely on a common neurocognitive system. Overall, these findings reveal a central, domain‐general system for artistic creativity, but with each domain relying to some degree on domain‐specific neural circuits.

## 1 INTRODUCTION

Over the past few decades, a growing number of studies have employed neuroimaging techniques to explore cognitive and brain mechanisms of creative and artistic performance. Despite some progress, however, the neural mechanisms of creativity remain incompletely defined. One reason relates to variability in how creativity is defined, leading to heterogeneity of measurement and evaluation across multiple domains of performance. Previous studies suggest that a wide range of brain regions are recruited during different creative thinking tasks, such as divergent thinking, artistic creativity, and insight, raising questions about the existence of a domain‐general neurocognitive system for creative thinking, beyond diffuse prefrontal activation (Dietrich & Kanso, 2010; Sawyer, 2011). On the other hand, recent reviews and research point to some consistent findings across different creative tasks and domains at the level of large‐scale brain network dynamics (Beaty, Benedek, Silvia, & Schacter, 2016), such as verbal creativity (e.g., divergent thinking) and artistic improvisation, which have shown similar patterns of functional connectivity between executive control network (ECN) and default network (DN). To some extent, these findings point to the possibility that creative thinking involves both domain‐general and domain‐specific neural resources. In this review, we aim to provide clarity on the contribution of such general and specific brain systems in diverse domains of artistic creativity.

### 1.1 Artistic creativity

Artistic creativity refers to an ability of individuals to produce novel, appropriate, and esthetically artistic products (Abraham, 2018), and it comprises a range of actions across different fields, such as music improvisation, drawing creativity, and literary creativity. All forms of artistic creativity reflects the expression of new ideas, in which the mental processing during artistic creation is related to the ability to connect weak and remote elements/concepts in a novel and appropriate way (Mednick, 1962). Given that all creative domains require the production of new ideas, it would seem plausible to identify common (i.e., domain‐general) brain systems by synthesizing neuroimaging findings across different fields of artistic creativity. This approach offers a promising means to clarify the core processes of creative thinking by exploring common and distinct neural correlates across diverse creative actions. Here, we focus on three domains of artistic creativity—musical creativity (improvisation), drawing creativity, and literary creativity. Neuroimaging research of creativity has focused on these domains of artistic performance because they are relatively amenable to the MRI scanner environment, and the output of participants can be collected and quantified using novel fMRI‐compatible equipment. Here, we use meta‐analytic neuroimaging to test whether each domain is supported by common and/or distinct brain regions.

#### 1.1.1 Musical creativity

Musical creativity mainly includes two forms: musical composition and musical improvisation (Deliège & Wiggins, 2006). Both involve the creation of novel and unique melodies, harmonies, and rhythms, within the constraints and conventions of a musical tradition (Brown, Martinez, & Parsons, 2006; Zatorre, Chen, & Penhune, 2007). In neuroimaging studies, musicians are typically asked to use MR‐compatible instruments to compose a novel melodic or rhythmic improvisation in real‐time, which is often compared to a condition requiring repeated performance of a familiar melody. Besides, jazz is one typical form of musical improvisation, in which musicians compose on the spot by assembling melodic, harmonic, and rhythmic elements (Limb & Braun, 2008). Several studies have shown that improvisation is associated with the right lateral prefrontal cortex (including the inferior frontal gyrus [IFG] and left dorsolateral prefrontal cortex [DLPFC]), supplementary motor area, premotor cortex, lateral temporal cortex, and insula (Bengtsson, Csikszentmihalyi, & Ullen, 2007; Brown et al., 2006; Villarreal et al., 2013). Some researchers emphasize that novel ideas during improvisations are mediated by deactivation of the lateral prefrontal cortex, reflecting reduced inhibitory control or self‐monitoring and presumably facilitating spontaneous cognition conducive to creative generation (Limb & Braun, 2008; Liu et al., 2012). In contrast, other studies on musical improvisation show increased prefrontal cortex engagement, including studies requiring musicians to improvise based on specific emotional cues (e.g., happy/fearful emotional content; Pinho, Ullen, Castelo‐Branco, Fransson, & de Manzano, 2016) and studies examining brain plasticity induced by long‐term musical training (Herholz & Zatorre, 2012; Pinho, de Manzano, Fransson, Eriksson, & Ullen, 2014). These discrepant prefrontal findings raise questions about the role of cognitive control in musical improvisation.

Investigations of musical improvisation have also focused on the role of expertise. Some researchers have suggested that improvising musicians may be better able to suppress stimulus‐driven attention and to exert less stringent evaluation during the creative process, thus expanding attentional scope and allowing more extraneous information to enter the processing system to produce novel melodies and rhythms (Berkowitz & Ansari, 2008; Pinho et al., 2014). For example, Pinho et al. (2014) found that the duration of improvisation experience was negatively associated with activity in prefrontal cortex (e.g., DLPFC). In addition, these same prefrontal regions showed increased functional connectivity with premotor areas, extending prior work linking prefrontal and premotor regions to melodic and rhythmic processing, respectively (de Manzano & Ullen, 2012). Taken together, the findings raise two possible interpretations regarding the role of lateral prefrontal cortex in musical creativity: one that emphasizes cognitive inhibition as a domain‐general cognitive process of creative thinking and another that emphasizes plastic effects as a domain‐specific process in creative actions.

#### 1.1.2 Drawing creativity

Drawing creativity, namely, visual artistic creativity, refers to the production of novel and esthetically‐pleasing visual‐forms (e.g., sketches, paintings, and graphic design) that depend upon visual mental imagery (Aziz‐Zadeh, Liew, & Dandekar, 2013; Dake, 1991). A case study conducted by Solso (2001) explored brain activity of a professional artist when sketching drawings of faces during fMRI. Compared to a single nonartist control subject, the professional artist exhibited lower activation in the right posterior parietal cortex, a region responsible for face processing, but higher activation in the right‐middle frontal area. Solso suggested that experts might dedicate more resources to high‐level cognitive processing for the “meaning” of faces rather than the “features” of faces. Recent studies have also found that creative drawing engages the right lateral prefrontal cortex (i.e., DLPFC). In this context, the DLPFC is thought to exert top‐down control over left lateral prefrontal cortex and posterior regions (e.g., parietal–temporal‐occipital area), suppressing interfering stimuli and supporting internal attention demands, visual imagination, and the integration of task‐relevant information during idea generation (Kowatari et al., 2009; Rominger et al., 2020). Other studies with nonprofessional painters have reported increased engagement of left frontal cortex during creative drawing (Aziz‐Zadeh et al., 2013; Huang et al., 2013; Saggar et al., 2015). Based on the twofold model of creativity, some researchers proposed that regions within the DN contribute to the generation of novel ideas in the early stages of drawing (Ellamil, Dobson, Beeman, & Christoff, 2012; Fan et al., 2014), whereas functional connectivity between the DN and the control network supports the evaluation of ideas in later phases (Beaty et al., 2016; Ellamil et al., 2012; Kleinmintz, Ivancovsky, & Shamay‐Tsoory, 2019).

Beyond the involvement of prefrontal cortex, several studies have shown that the medial temporal lobe (MTL), including hippocampus, parahippocampus, and fusiform gyrus, exhibits greater recruitment during drawing creativity, such as visual art design (Ellamil et al., 2012; Fan et al., 2014; Hahm, Kim, Park, & Lee, 2017; Kowatari et al., 2009; Park, Kirk, & Waldie, 2015). The fusiform gyrus typically activates during tasks involving visual imagery, a form of mental representation characterized by internal sensory imagination and subjective experiences in the absence of external stimuli (Winlove et al., 2018). With respect to creativity, there is increasing focus on the link between creative thinking and activation in memory‐related regions within MTL. For example, Ellamil et al. (2012) suggested that, during creative idea generation, MTL activation may reflect the construction of new associations that rely on the retrieval and integration of semantic and episodic representations. Moreover, recent research has found that an episodic specificity induction—an experimental procedure that promotes an episodic retrieval orientation—can improve divergent creative thinking performance (Madore et al., 2015) by increasing activation in left anterior hippocampus (Madore, Addis, & Schacter, 2015; Madore, Thakral, Beaty, Addis, & Schacter, 2017), further implicating MTL regions in creative thinking.

#### 1.1.3 Literary creativity

Literary creativity has been investigated with fMRI in the contexts of story production and poetry creation. Early research suggested that creative language usage differs from canonical language processing with respect to brain lateralization. For example, Howard‐Jones et al. reported activation of bilateral medial frontal gyri, left middle frontal gyrus, and anterior cingulate cortex during a story generation task. The authors interpreted this activation pattern as reflecting increased episodic memory retrieval, maintaining more possibilities in working memory, and monitoring/evaluation to achieve more appropriate and novel criteria Howard‐Jones, Blakemore, Samuel, Summers, & Claxton, 2005). Several recent neuroimaging studies have found that creative writing is associated with a wide range of brain regions, with hemispheric dominance effects depending on the experimental conditions and the subject's level of expertise (Chen et al., 2019; Erhard, Kessler, Neumann, Ortheil, & Lotze, 2014). For example, compared to inexperienced writers, professional writers recruited stronger activation within the DN, as well as regions involved in memory retrieval and emotion processing (Erhard et al., 2014; Liu et al., 2015). In line with studies on drawing creativity, studies of poetry composition have reported decreased coupling of default and control network regions during poetry generation but increased coupling between the networks during poetry evaluation (Liu et al., 2015), consistent with neuroimaging investigations on domain‐general creativity using a verb generation task (Beaty, Christensen et al., 2017). Together, these findings are in line with recent theories of creativity that emphasize dynamic interactions between the default and control networks (Beaty et al., 2016; Kleinmintz et al., 2019; Shi et al., 2018). However, the extent to which these networks support domain‐general versus domain‐specific creative performance remains unclear.

### 1.2 The present study

Although different art forms require distinct domain‐specific skills, knowledge, and instruments, they all share some domain‐general creative demands, such as the production of novel elements, unconventional performance, and overcoming fixation (Abraham, 2018). The contribution of domain‐general processes raises the central question of whether different artistic domains rely on common neural substrates, and whether these commonalities correspond to similar cognitive process during creative processes. On the one hand, extant studies and reviews point to considerable heterogeneity across different modalities of creative performance (e.g., verbal, visuospatial, and musical improvisation). On the other hand, there seems to be some overlap in the large‐scale brain networks associated with creative thinking across several different domains (Beaty, 2015; Boccia, Piccardi, Palermo, Nori, & Palmiero, 2015; Pidgeon et al., 2016; Wu et al., 2015). Another question concerns the domain‐specificity of neural recruitment for each creative domain, that is, which brain circuits are unique to creative performance in literary, musical, and drawing creativity. Although prior meta‐analyses have considered creative domains (Pidgeon et al., 2016; Wu et al., 2015), such meta‐analyses have tended to lack specificity, often including a wide range of creative tasks and domains in a single analysis (Boccia et al., 2015). To date, a systematic meta‐analysis that synthesizes studies within specific artistic domains, quantifying neural consistencies and differences among different forms of creativity, has not been conducted. Fortunately, due to the increasing interest in the neuroscience of creativity, we now have access to a sufficient pool of studies within each artistic domain for meta‐analytic inquiry. In the present meta‐analysis, we can thus ask the question of how the brain achieves creative performance across diverse artistic forms, providing insight into longstanding debates regarding the domain‐specificity and generality of creativity.

Here, we conducted activation likelihood estimation (ALE) meta‐analyses on three artistic creative modalities: music, drawing, and literary. First, we applied ALE meta‐analyses to identify brain activation patterns for each creative modality. Then, we conducted conjunction and contrast analyses of these meta‐analytic maps to assess the common and distinct neural correlates supporting the three artistic forms. In terms of previous fMRI meta‐analyses on verbal and visuospatial creative thinking, as well as relevant reviews (Boccia et al., 2015; Pidgeon et al., 2016; Wu et al., 2015), we hypothesized that the neural correlates across the three artistic modalities would mainly converge in the lateral prefrontal cortex (PFC), the SMA, and some medial regions of the DN. Furthermore, we hypothesized that distinct activation would mainly be observed in regions related to domain‐specific demands for each form, such as the SMA for motor planning in music improvisation, the occipitotemporal gyrus for visual imagination in drawing, and the lateral temporal cortex for semantic processes in literary creativity.

## 2 METHODS

### 2.1 Literature selection and exclusion criteria

A systematic literature search was carried out using PubMed and Web of Knowledge databases for peer‐reviewed fMRI and positron emission tomography (PET) studies on artistic creativity up to July 14th, 2019. Three artistic modalities involving creativity were included in the meta‐analysis: musical creativity, drawing creativity, and literary creativity. For each modality, all relevant search terms were combined (“AND”) with “fMRI” or “PET.” Specifically, the keywords for musical creativity included “musical improvisation,” “musical creativity,” “music creativity,” and “compose AND creativity.” This search yielded a total of 140 studies; after removing duplicates, 110 studies were retained. For drawing creativity, the keywords included “drawing creativity,” “visual–spatial creativity,” “visual creativity,” “visual divergent thinking,” and “figural creativity.” In addition, one fMRI study published using Chinese‐language was included. This search yielded a total of 128 studies; after removing duplicates, 93 studies were retained. For literary creativity, the keywords included “creative writing,” “poetry,” “story and creativity,” “writing and divergent thinking,” and “metaphor creativity.” This search yielded a total of 103 studies; after removing duplicates, 73 studies were retained.

**FIGURE 1:**
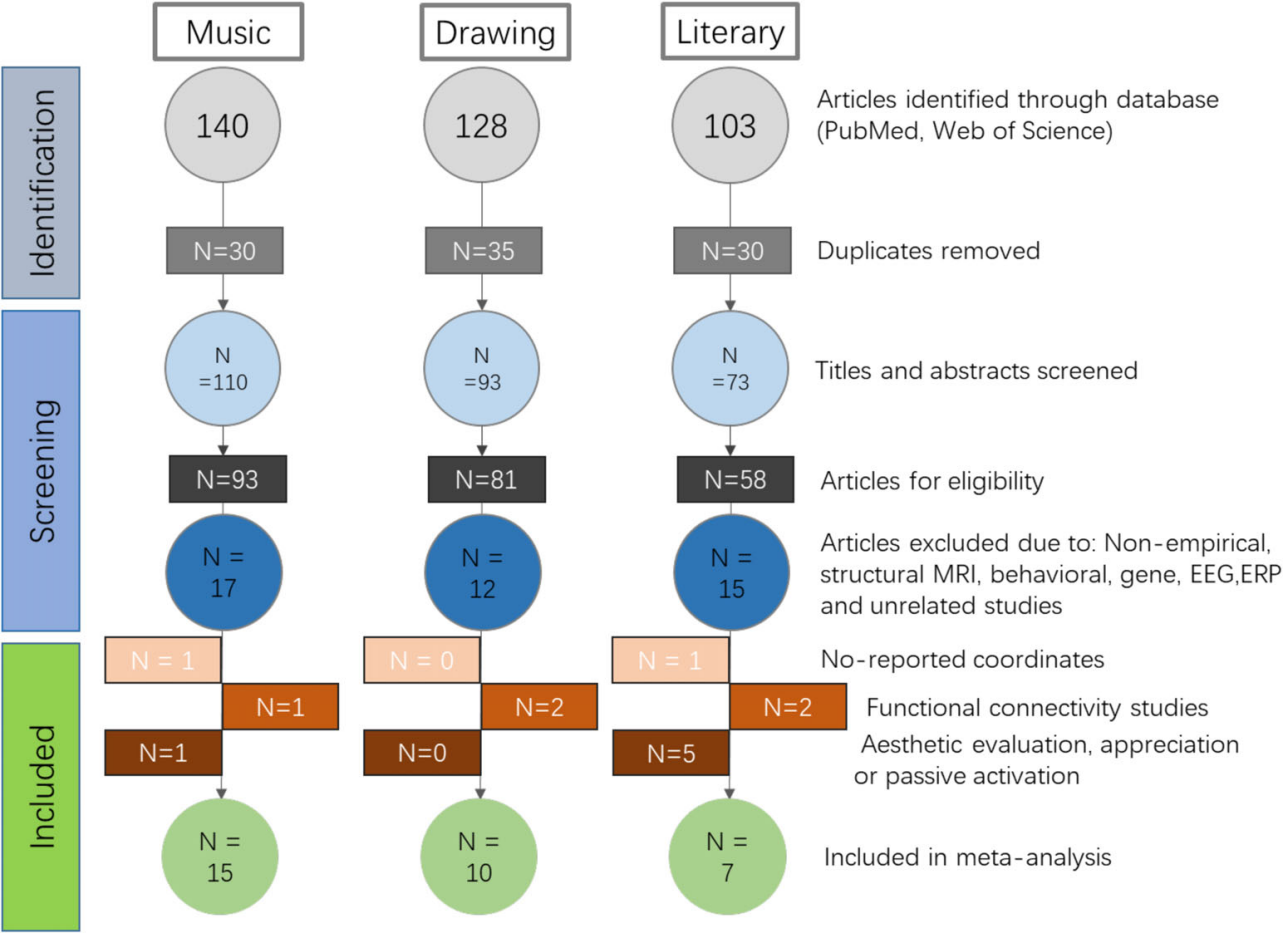
Flowchart illustrating literature selection and exclusion in the meta‐analysis

Further inclusion criteria for these candidate studies were the following (see Figure 1): (a) Studies were empirical and used task‐fMRI/PET approaches; studies employing EEG, gene analysis, and structural MRI were thus excluded. (b) Studies reported three‐dimensional coordinates in standard space (i.e., Montreal Neurological Institute [MNI] or Talairach) and results with whole‐brain analyses (Eickhoff et al., 2009). (c) The results for those articles derived from functional connectivity analyses were excluded. (d) Experimental paradigms required active task engagement (e.g., thinking, writing, and improvising); studies on esthetic evaluation (e.g., poem/artwork/music appreciation) were excluded.

After applying these exclusion criteria, a total of 32 separate studies were included in the ALE meta‐analysis. Musical creativity studies included 253 foci from 21 experiments with 291 participants (30.34% females, average age = 30.81 ± 6.7) across 15 studies; drawing creativity studies included 210 foci from 13 experiments with 252 participants (58.03% females, average age = 23.57 ± 3.26) across 10 studies; and literary creativity included 169 foci from 14 experiments with 199 participants (19.25% females, average age = 24.62 ± 3.65) across seven studies. More details of the included literature can be seen in Table 1.

**TABLE 1:** Summary of studies and contrasts included in the meta‐analysis

Study	N(F/M)	Scanner	Type	Task	Contrasts	Foci
Shah et al. (2013)	28 (14/14)	fMRI	Literary	Creative writing	Brainstorming > copying; creative writing > copying	6
Bechtereva et al. (2004)	25 (0/25)	PET	Literary	Story generation	Difficult > easy; difficult > reading; difficult > words	15
Howard‐Jones et al. (2005)	8 (7/1)	fMRI	Literary	Story generation	Creative story > uncreative story	4
Benedek, Jauk, et al. (2014)	28 (18/10)	fMRI	Literary	Metaphor production	Creative metaphor > literal generation	8
Beaty, Silvia, et al. (2017)	35 (22/13)	fMRI	Literary	Metaphor production	Metaphor > synonym	12^a^
Erhard et al. (2014)	48 (22/26)	fMRI	Literary	Creative writing	Brainstorming > copying; creative writing > copying	33
Liu et al. (2015)	27 (15/12)	fMRI	Literary	Poetry composition	Generate new poem > recite memorized poems	41
Aziz‐Zadeh et al., 2013)	13 (7/6)	fMRI	Drawing	Shapes assembling	Visual imagery > control	10
Cai et al. (2018)	16 (8/8)	fMRI	Drawing	Shapes assembling	Visual imagery > control	12
Ellamil et al. (2012)	15 (9/6)	fMRI	Drawing	Cover illustrations	Generation > evaluation	46
Fan et al. (2014)	23 (17/6)	fMRI	Drawing	Face design task	Unrestricted design > restricted design	9
Hahm et al. (2017)	25 (14/11)	fMRI	Drawing	Figural TTCT	Drawing imagery > line tracking	31
Huang et al. (2013)	26 (15/11)	fMRI	Drawing	Construct image	Creative > uncreative	3
Kowatari et al. (2009)	40 (24/16)	fMRI	Drawing	Designing new pens	Creative design > counting	19
Park et al. (2015)	48 (31/17)	fMRI	Drawing	Figural TTCT	Creative drawing > line tracking	12
Saggar et al. (2017)	36 (18/18)	fMRI	Drawing	Figural pictionary task	Word‐drawing > zigzag‐drawing	31
Saggar et al. (2015)	30 (16/14)	fMRI	Drawing	Figural pictionary task	Word‐drawing > zigzag‐drawing	34
Villarreal et al. (2013)	24 (15/9)	fMRI	Music	Generate rhythm	Create > repeat	6
Pinho et al. (2016)	39 (15/24)	fMRI	Music	Improvisation	Pitch‐set > rest	13
Pinho et al. (2014)	39 (15/24)	fMRI	Music	Improvisation	Improvisation > rest	4
McPherson et al. (2016)	12 (1/11)	fMRI	Music	Improvisation	Improvisation > chromatic	12
Lu et al. (2017)	29 (15/14)	fMRI	Music	Improvisation	Improvisation > random button press	41
Liu et al. (2012)	12 (0/12)	fMRI	Music	Freestyle rap	Improvised > conventional	19
Limb & Braun (2008)	6 (0/6)	fMRI	Music	Jazz improvisation	Improvised > control	31
Donnay et al. (2014)	11 (0/11)	fMRI	Music	Jazz improvisation	Improvised > control	36
Dhakal et al. (2019)	24 (0/24)	fMRI	Music	Vocalize and imagine improvised	Improvised > prelearned	20
de Manzano & Ullen (2012)	18 (1/17)	fMRI	Music	Improvisation	Improvised > sight‐reading	8
de Manzano & Ullen (2012)	15 (14/1)	fMRI	Music	Improvisation	Melodic and rhythmic improvisation > rest	4
Brown et al. (2006)	10 (5/5)	fMRI	Music	Improvisation	Melodic improvisation > rest	40
Berkowitz & Ansari (2008)	13 (5/8)	fMRI	Music	Improvisation	Improvised > familiar patterns	20
Berkowitz & Ansari (2010)	28 (15/13)	fMRI	Music	Improvisation	Melodic improvisation > patterns conditions	1
Bengtsson et al. (2007)	11 (0/11)	fMRI	Music	Improvisation	Improvised > reproduce	14

a Foci was identified using multivariate pattern analysis.

### 2.2 Activation likelihood estimation

To identify consistent brain activation for each style of artistic creativity, three separate meta‐analyses were conducted using the latest GingerALE (version 3.0.2, http://brainmap.org), which is a freely available and quantitative meta‐analysis method (Eickhoff et al., 2009; Turkeltaub et al., 2012). GingerALE relies on ALE, which compares foci compiled from multiple articles and estimates the magnitude of overlap, yielding clusters most likely to become active across studies. The most recent algorithm minimizes within‐group effects and provides increased power by allowing for the inclusion of all possible relevant experiments (Eickhoff, Laird, Fox, Lancaster, & Fox, 2017; Turkeltaub et al., 2012). Before ALE, coordinates reported in Talairach space were translated into the coordinates to MNI space using the convert Foci embedded within GingerALE. Statistical maps were thresholded at *p* < .05 using a family‐wise error‐correction at the cluster level, corrected for multiple comparisons (5,000 permutations) with a cluster forming threshold of *p* < .001 (Eickhoff et al., 2017). To investigate the common regions across three artistic creative modalities, we used the “image calculator” function in SPM8 to calculate areas with equal activation likelihood, which is equivalent to identifying the intersection for the resultant maps from three separate meta‐analyses.

To compare the results of pairwise meta‐analysis (e.g., music vs. drawing, drawing vs. literary, and music vs. literary), we also performed conjunction analyses and contrast analyses in GingerALE. Due to the exploratory nature of the analysis, the correction for pairwise contrast analyses was loosely defined at an uncorrected *p* < .005 with 5,000 permutations and a minimum cluster size of 10 mm^3^.

### 2.3 Results visualization

All significant clusters were reported, including the volume, coordinates in MNI space, and Z‐scores at peaks. For visualization purposes, these results were registered onto an MNI‐space template (i.e., Colin27_T1_seg_MNI.nii) brain using Mango (ric.uthscsa.edu/mango) and MRIcron software (http://www.sph.sc.edu/comd/rorden/mricron).

## 3 RESULTS

### 3.1 Regions of activation in music creativity

**FIGURE 2:**
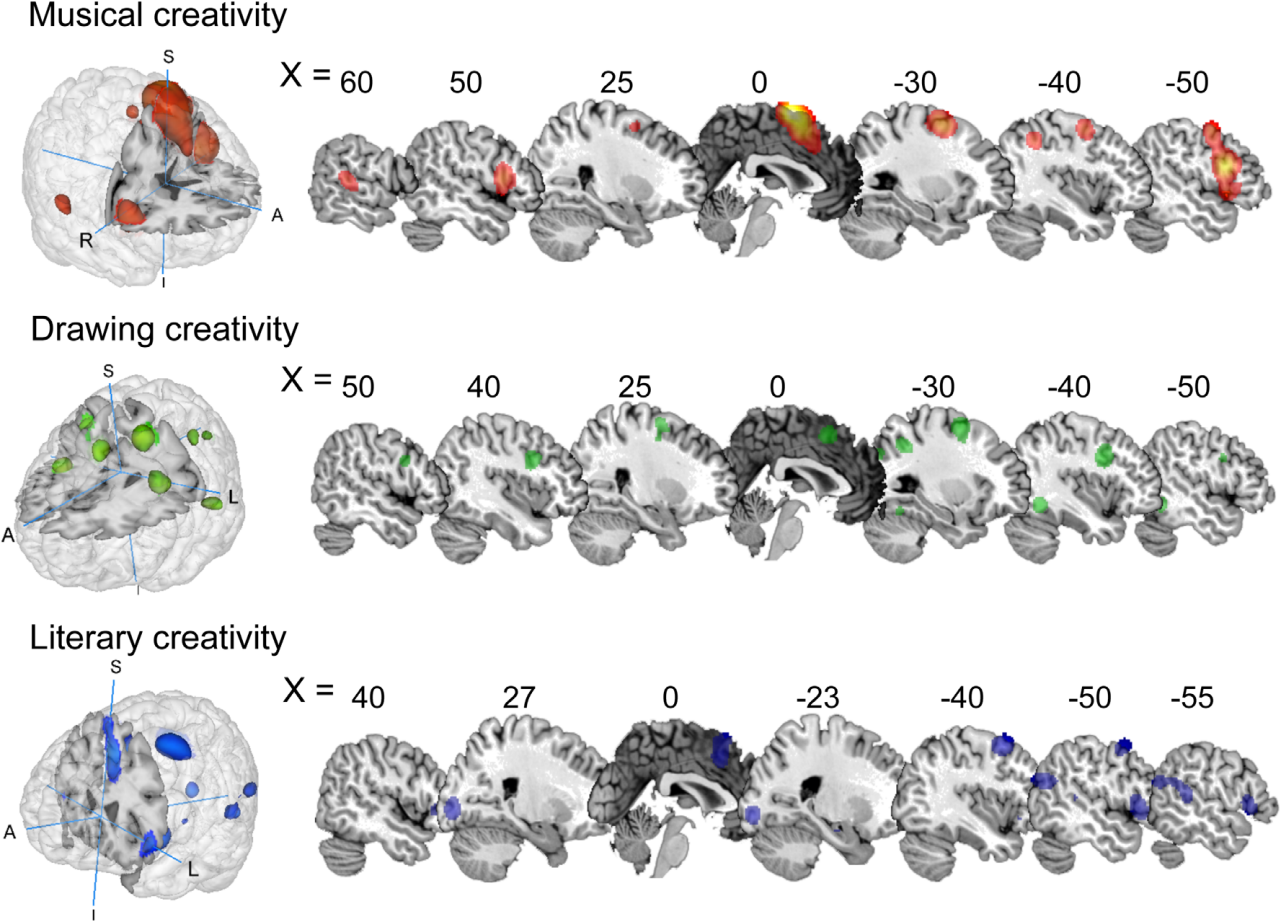
Results of single ALE meta‐analysis for each artistic creativity

Neuroimaging studies of musical creativity exclusively focused on musical improvisation (melodic, rhythmic, and jazz), and they required performance on MR‐compatible instruments during functional imaging. A meta‐analysis of 21 musical creativity experiments showed a subset of activated clusters associated with musical improvisation (see Figure 2a and Table 2), including the bilateral SMA extending to medial prefrontal cortex (mPFC), precentral gyrus (PreCG), superior frontal gyrus (SFG), and middle frontal gyrus (MFG); bilateral inferior frontal gyrus (IFG), left inferior parietal lobule (IPL), and the right superior temporal gyrus (STG).

**TABLE 2:** Results of meta‐analyses for each artistic type

Cluster	Anatomical labels	R/L	BA	MNI coordinates				
				*X*	*Y*	*Z*	ALE *Z*‐value	Volume (mm^3^)
Music creativity > control								
1	Supplementary motor area extending medial frontal gyrus, precentral gyrus, superior frontal gyrus, middle frontal gyrus, and inferior frontal gyrus	L	6/9/32/44/24	−4	4	66	8.3	64,224
2	Inferior frontal gyrus	R	13/44/45	52	14	8	4.71	5,480
3	Superior temporal gyrus	R	22/41/42/21	60	−32	8	4.02	2,280
4	Inferior parietal lobule	L	40	−38	−46	42	4.28	1944
Drawing creativity > control								
1	Inferior frontal gyrus	R	9	46	8	26	4.96	3,736
2	Middle frontal gyrus	L	6	−26	0	56	4.65	3,616
3	Precentral gyrus	L	6	−40	4	30	4.33	3,520
4	Fusiform gyrus	L	37	−42	−54	−12.7	4.28	3,160
5	Supplementary motor area	L	6	−2	12	48	4.4	2,848
6	Inferior parietal lobule	L	40	−28	−54	40	4.09	2088
7	Middle frontal gyrus	R	6/32	26	−6	58	3.81	1968
8	Superior occipital gyrus	L	31	−24	−78	32	3.79	1,680
Literary creativity > control								
1	Supplementary motor area	L	6/32	−6	18	42	5.61	9,424
2	Middle temporal gyrus/superior temporal gyrus	L	39	−50	−66	22	4.1	5,040
3	Middle frontal gyrus	L	6	−40	8	52	4.9	4,976
4	Inferior frontal gyrus	L	44	−50	18	0	4.37	3,576
5	Lingual gyrus	L	18	24	−90	−2	4.56	2,784
6	Lingual gyrus	L	18	−22	−92	−6	4.57	2,280
7	Cerebellum posterior lobe	R	\	8	−70	−22	3.7	912
8	Inferior frontal gyrus	R	13/45	48	28	−2	3.44	384
9	Parahippocampal gyrus	L		−24	−12	−20	3.27	192
10	Medial dorsal thalamic nucleus	L	\	−12	−18	12	3.21	88

*Note*: These presented clusters were thresholded at *p* < .05 using a family‐wise error‐corrected at cluster level for multiple comparisons (5,000 permutations).

### 3.2 Regions of activation in drawing creativity

The analysis of drawing creativity revealed a set of significant activations associated visual imagery and motor control, including a large cluster in the left hemisphere comprised of SMA, MFG, PreCG, Fusiform Gyrus (FG), IPL, and superior occipital gyrus (SOG); two clusters were also found in the right hemisphere: MFG and IFG (see Figure 2b and Table 2).

### 3.3 Regions of activation in literary creativity

For literary creativity, several expressive forms were included in the meta‐analysis, including story generation, novel metaphor production, and poetry composition. Results revealed a set of significant clusters in the left hemisphere, including the SMA, middle temporal gyrus (MTG; extending to STG and MFG), IFG, lingual gyrus, parahippocampal gyrus, and medial dorsal nucleus; two clusters were also found in the right hemisphere: IFG and cerebellum posterior lobe (see Figure 2c and Table 2).

### 3.4 Domain‐general regions across three domains of artistic creativity

**FIGURE 3:**
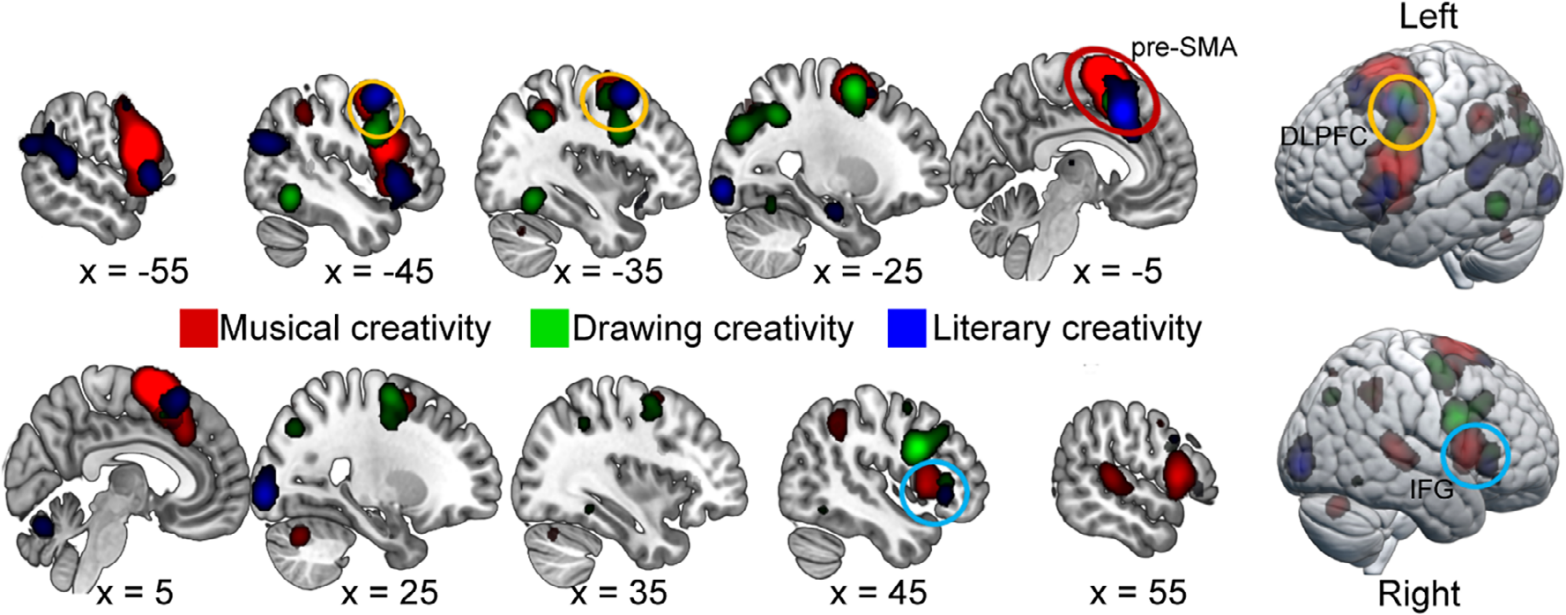
Brain regions showing significant common activation across musical creativity, drawing creativity, and literary creativity

The conjunction analysis showed commonly activated regions across musical, drawing, and literary creativity including the left pre‐SMA (*x* = −8, *y* = 14, *z* = 38; cluster size = 549 voxels), left DLPFC (*x* = −44, *y* = 2, *z* = 42; cluster size = 148 voxels), and right IFG (*x* = 42, *y* = 26, *z* = 0; cluster size = 18 voxels; see Figure 3).

### 3.5 Domain‐specific regions across three domains of artistic creativity

**FIGURE 4:**
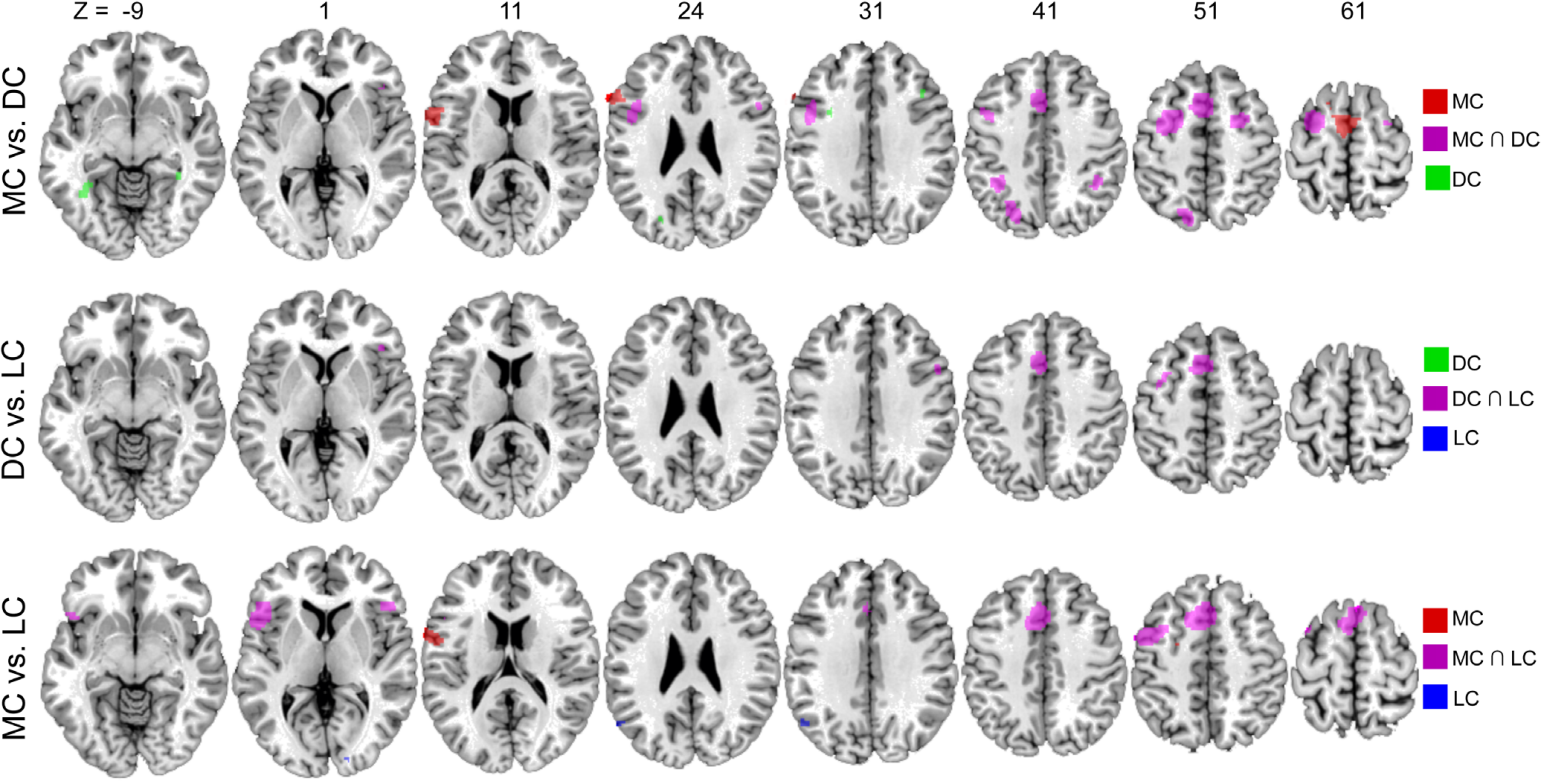
Brain regions showing common and distinct activation between any two artistic creativity types. DC, drawing creativity; LC, literary creativity; MC, musical creativity; R/L, right/left; these presented clusters were thresholded at uncorrected *p* < .005 with 5,000 permutations and a minimum cluster size of 10 mm^3^

Conjunction analyses between musical creativity and drawing creativity revealed consistent activation in the left SMA, bilateral MFG, left precuneus, bilateral IFG, and bilateral IPL. Compared to drawing creativity, musical creativity showed stronger activation in the SMA and bilateral IFG; the reverse contrast showed stronger activation for drawing creativity in the left fusiform gyrus, right parahippocampal gyrus, right MFG, and left precuneus (see Figure 4a and Table 3).

**TABLE 3:** Conjunction and contrast analyses between two artistic types

Cluster	Anatomical labels	R/L	BA	MNI coordinates			ALE *Z*‐value	Volume (mm^3^)
				*X*	*Y*	*Z*		
MC ∩ DC								
1	Middle frontal gyrus	L	6/9	−26	0	56	0.0038	9,432
2	Supplementary motor area	L	6	−2	12	48	0.0036	5,246
3	Precuneus	L	7	−18	−17	46	0.0026	2,264
4	Middle frontal gyrus	R/L	6	30	0	56	0.0027	2,112
5	Inferior parietal lobule	L	40	−34	−48	38	0.0029	1,000
6	Inferior parietal lobule	R	40	40	−46	40	0.0025	600
7	Inferior frontal gyrus	R	9	48	12	20	0.0026	368
8	Inferior frontal gyrus	R	13	44	26	4	0.0025	344
MC > DC								
1	Supplementary motor area	L	6	−1	−2	64	3.29	2,704
2	Inferior frontal gyrus	L	6/44	−58	3	9	3.29	1,056
3	Inferior frontal gyrus	R	9	−60	21	25	3.29	1,024
4	Supplementary motor area	L	6	−14	10	60	3.29	56
DC > MC								
1	Fusiform gyrus	L	37	−33	−47	−13	3.29	648
2	Parahippocampal gyrus	R	37	36	−40	−10	3.29	96
3	Middle frontal gyrus	R	9	40	23	30	3.09	48
4	Precuneus	L	31	−23	−76	24	3.09	32
DC ∩ LC								
1	Supplementary motor area	L	6/32	−4	14	46	0.0035	4,392
2	Middle frontal gyrus	L	6	−32	6	56	0.0025	1,240
3	DLPFC	R	9	52	12	32	0.0021	224
4	Inferior frontal gyrus	R	13	46	28	2	0.0022	192
5	Middle frontal gyrus	L	6	−38	0	46	0.0021	8
DC > LC	None							
LC > DC	None							
MC ∩ LC								
1	Supplementary motor area	L	6/32	−6	18	44	0.0043	16,752
2	Inferior frontal gyrus	L	44/13/47	−50	18	−2	0.0032	4,920
3	Inferior frontal gyrus	R	45/13/47	48	26	0	0.0024	648
MC > LC								
1	Precentral gyrus	L	6/4	−58	−3	15	3.35	784
2	Middle frontal gyrus	L	\	−24	−4	47	3.09	16
LC > MC								
1	Angular	L	39/19	−52	−66	30	3.54	264
2	Lingual gyrus	R	17	18	−95	3	3.16	64

*Note*: DC, drawing creativity; LC, literary creativity; MC, musical creativity; R/L, right/left. These presented clusters were thresholded at uncorrected *p* < .005 with 5,000 permutations and a minimum cluster size of 10 mm^3^.

The conjunction of drawing creativity versus literary creativity showed common regions for the two artistic domains in left SMA, left MFG (including DLPFC), right DLPFC, and right IFG. No significant activation differences were found between drawing creativity and literary creativity (see Figure 4b and Table 3).

Common activation of musical creativity and literary creativity was found in the left SMA and bilateral IFG. Musical creativity showed stronger activation in the left PreCG and left MFG, whereas the reverse contrast showed activation of the left angular and right lingual gyrus (see Figure 4c and Table 3).

## 4 DISCUSSION

In the present meta‐analysis, we first sought to identify common brain regions associated with three domains of artistic creativity (i.e., music, drawing, and literary creativity). Second, we aimed to identify distinct regions with respect to each creative domain. Overall, the results of the meta‐analyses revealed three prefrontal brain regions common to music, drawing, and literary creativity: left pre‐SMA, left DLPFC, and right IFG. Moreover, contrasting these modalities revealed reliable domain‐specific activation for each artistic domain: music creativity (improvisation) recruited bilateral IFG, left PreG, and left MFG compared to the other two artistic modalities; drawing creativity recruited the right MFG, left fusiform gyrus, left precuneus, and right parahippocampal gyrus compared to musical creativity; and literary creativity recruited the left angular and right lingual gyrus compared to musical creativity. Together, these meta‐analytic findings indicate that a set of prefrontal brain regions support creative performance across diverse artistic domains, but that specific regions also support creativity in each creative domain. In the following sections, we discuss the potential functional significance of these converging findings for understanding artistic creativity, focusing on current theories of creativity as a domain‐general process.

### 4.1 Neuroscience mechanisms of commonality in artistic creativity

#### 4.1.1 Pre‐SMA

The SMA was found to be consistently activated, with robust overlap across three canonical forms of artistic creativity. Although previous literature indicates that the SMA is actively engaged during musical improvisation, novel drawing, and literary creativity (Erhard et al., 2014; Pinho et al., 2014; Shah et al., 2013), previous work has emphasized its possible role in artistic performance because SMA is a heterogeneous region implicated in diverse cognitive functions (Cona & Semenza, 2017). The medial region of Brodmann area 6 can be subdivided into the SMA‐proper and the anterior SMA (pre‐SMA) by the vertical line (MNI space, *y* = 0) crossing the anterior commissure (Kim et al., 2010), in which SMA‐proper is predominantly linked to motor‐related functions and pre‐SMA is more linked to higher‐order cognitive control (Hertrich, Dietrich, & Ackermann, 2016). Previous evidence indicated that pre‐SMA is richly connected to IFG, AnG, anterior cingulate cortex, as well as subcortical regions, such as the striatum (Chouinard & Paus, 2010; Johansen‐Berg et al., 2004; Kim et al., 2010; Lu, Preston, & Strick, 1994); these regions are important for higher‐order aspects of action, such as integrating information about action plans, motivation, and inhibitory control (Lima, Krishnan, & Scott, 2016). In the present meta‐analysis, the overlapping region of SMA (*y* = 8) across three artistic domains belonged to the pre‐SMA. We thus propose that pre‐SMA may also be involved in higher‐order cognitive processes during artistic creativity involved in planning and selecting complex action sequences (Beaty, 2015).

Earlier work on music improvisation has consistently found that pre‐SMA activity scales with sequence complexity, implicating pre‐SMA in internally‐driven sequence selection (Bengtsson et al., 2007; Berkowitz & Ansari, 2008; Brown et al., 2006; Lau, Rogers, Ramnani, & Passingham, 2004), which is critical for the free generation of rhythmic and melodic structures during music improvisation (de Manzano & Ullen, 2012). In addition, recent studies have implicated pre‐SMA in motor imagery (e.g., auditory imagery and mental rotation; Lima et al., 2016) and higher‐level planning processes (e.g., motor planning; (Picard & Strick, 1996; Winstein, Grafton, & Pohl, 1997)—potentially facilitating the generation, manipulation, and selection of spontaneous behavioral responses required for creative action (Dhakal, Norgaard, Adhikari, Yun, & Dhamala, 2019; Lu et al., 2017; Villarreal et al., 2013). Aziz‐Zadeh et al. (2013) pointed out that the pre‐SMA is often recruited across various creative domains, including music, language, and drawing, but that the region also interacts with other prefrontal regions, such as DLPFC and IFG, to support creative performance. This observation is consistent with several studies on functional connectivity implicating the interaction of a wide range of frontal regions with pre‐SMA (Pinho et al., 2016). Taken together, we propose that the pre‐SMA plays a crucial role in domain‐general processes across artistic creativity, including internally‐driven free selection of motor sequences, mental imagination, and motor planning, potentially through its interaction with other prefrontal brain regions.

#### 4.1.2 Left DLPFC

Meta‐analytic results also implicated the left DLPFC as a core domain‐general region supporting artistic creativity. The DLPFC is consistently involved in a range of cognitive processes associated with executive function, such as working memory, flexible attention, and decision making (Bishop, 2009; Curtis & D'Esposito, 2003; Hare, Camerer, & Rangel, 2009). In previous MRI studies on creativity, the DLPFC has been recruited during various creative tasks, including verbal divergent thinking (Abraham et al., 2012; Beaty, Benedek, Kaufman, & Silvia, 2015; Howard‐Jones et al., 2005; Sun et al., 2016), insight problem solving (Tik et al., 2018), musical improvisation (Bengtsson et al., 2007; Berkowitz & Ansari, 2008), scientific problem solving (Tong et al., 2013), and visuospatial creativity (Aziz‐Zadeh et al., 2013; Gilbert, Zamenopoulos, Alexiou, & Johnson, 2010; Kowatari et al., 2009). The DLPFC may support creativity via flexible attention and working memory, serving to maintain and update relevant information across diverse creative domains.

Although the specific contribution of DLPFC to creative thinking remains debated, two emerging views have been suggested. One view is that DLPFC functions to evaluate and select candidate ideas through deliberate and analytic information processing (Howard‐Jones & Murray, 2003). The twofold model of creativity posits that creative thinking depends on a dynamic cycle between idea generation and evaluation (Finke, Ward, & Smith, 1992); here, the DLPFC—as a key region of the ECN—plays an important role during the evaluation phase, in which candidate ideas receive valuation, monitoring, and selection prior to creative output (Kleinmintz et al., 2019). Moreover, an increasing number of studies also indicate that highly creative individuals showed stronger connectivity between this “evaluation network” (ECN) and a second network that supports idea generation (i.e., the DN; Beaty et al., 2016; Zhu et al., 2017). In one study of drawing creativity, for example, idea generation was found to be related to widespread DN activation, whereas idea evaluation showed more co‐activation of both DN and ECN—including the DLPFC and dACC—implying the engagement of cognitive control and meta‐cognitive evaluative processing (Ellamil et al., 2012).

Another view of DLPFC's role in creativity has emphasized goal maintenance. According to this view, DLPFC activation during creative performance reflects the maintenance of higher‐order task goals and constraints, thereby facilitating the novelty and appropriateness of creative output. One common goal across creative tasks and domains is the goal to think creatively; indeed, most tasks explicitly instruct participants to “be creative” when generating novel products or ideas. This goal‐directed requirement to “be creative” can thus act like an anchor to guide cognitive processing during the entire task. For example, research on visual creativity found that creative design, compared to a control task, showed more activity in the left DLPFC, potentially attributed to goal‐directed planning of novel solutions by a top‐down direction of the creative process (Aziz‐Zadeh et al., 2013; Saggar et al., 2015). In a similar vein, a music improvisation study in professional pianists found that the activity and connectivity of the DLPFC strongly relied on the task constraints. Specifically, in a condition that specified the piano notes that could be used for improvisation (i.e., pitch set)—requiring the maintenance of the goal to restrict performance to specific notes—the DLPFC showed increased coupling with the bilateral dorsal promotor and the SMA. Conversely, in a condition that asked participants to improvise based on a specific emotion—requiring the maintenance of the goal to tailor performance to express a given emotion—the DLPFC showed increased coupling with several regions of the DN (Pinho et al., 2016). These findings suggest that DLPFC activity may be predominantly related to maintaining and integrating goal‐relevant information during creative performance (Beaty et al., 2016) as well as other central executive functions, such as flexible attention and selection (Kenett et al., 2018).

#### 4.1.3 Right IFG

Neuroimaging research on domain‐general creativity (e.g., verbal divergent thinking) has consistently implicated the ventrolateral PFC (i.e., IFG; e.g., Abraham et al., 2012; Benedek, Beaty, et al., 2014; Benedek, Jauk, et al., 2014; Fink et al., 2009; Zhu, Zhang, & Qiu, 2013). The IFG is subdivided into opercular (dorsal), triangular (middle), and orbital (ventral) regions, each with distinct functional roles (Aron, Robbins, & Poldrack, 2004, 2014; Costafreda et al., 2006; Desikan et al., 2006). In the context of research on creative thinking, converging evidence suggests that the left IFG supports controlled semantic retrieval and selection (Badre & Wagner, 2007). The ventral IFG, in particular, supports the controlled retrieval of information derived from semantic and episodic memory systems, thus potentially boosting the retrieval of relatively weak semantic associations (Badre, Poldrack, Paré‐Blagoev, Insler, & Wagner, 2005; Barredo, Öztekin, & Badre, 2013; Ralph, Jefferies, Patterson, & Rogers, 2017). Likewise, neuroimaging studies on memory control indicated that the right IFG is implicated in suppression of interfering memories during retrieval, which is critical for idea generation and the concomitant inhibition of prepotent ideas that lack originality. Aron et al. (2014) proposed that the right IFC and associated networks (i.e., prefrontal–basal ganglia network) can be viewed as a “brake” implementing inhibitory control in various modes (e.g., response inhibition, task‐set switching, and memory retrieval, etc.), and in different contexts (external and internal triggers; Aron et al., 2014). Although the right IFG serves broad inhibitory functions, as well as other executive control processes (such as attentional detection or monitoring), activation of this region during creative performance is consistent with the inhibitory demands of creative tasks, which can require the suppression of obvious thoughts and prepotent responses to create something new.

Likewise, previous studies on artistic creativity have pointed to a right‐hemispheric dominance in the PFC, particularly for experts (Bhattacharya & Petsche, 2002; Chen et al., 2019; Miller & Cohen, 2001). Kowatari et al. (2009) indicated that professional design training may facilitate inhibitory control of unwanted information via right PFC (including IFG) compared to left PFC (Kowatari et al., 2009). This inhibitory view was supported by lesion studies and evidence from brain‐stimulation. For example, patients with damage to left IFG showed higher scores on divergent thinking tasks (Mayseless, Aharon‐Peretz, & Shamay‐Tsoory, 2014; Shamay‐Tsoory, Adler, Aharon‐Peretz, Perry, & Mayseless, 2011). Likewise, inhibitory brain stimulation (tDCS, TMS, and tACS) targeting left IFG was found to increase originality scores (Ivancovsky, Kurman, Morio, & Shamay‐Tsoory, 2019; Kleinmintz et al., 2018; Lustenberger, Boyle, Foulser, Mellin, & Fröhlich, 2015). Recent research found that anodal right IFG stimulation coupled with cathodal tDCS over the left IFG facilitates novel idea production, whereas the reverse stimulation does not (Mayseless & Shamay‐Tsoory, 2015). These converging lines of evidence suggest that the right IFG may act as a regulator in controlled semantic retrieval, dampening interfering information by balancing its engagement in concert with the left IFG.

### 4.2 Neuroscience mechanisms of differentiation in artistic creativity

The current meta‐analysis revealed several domain‐specific regions supporting creative performance across studies of music, drawing, and literary creativity. Compared to drawing creativity and literary creativity, musical creativity was associated with more widespread frontal activity including SMA‐proper, PreG, left IFG, and left MFG. Based on prior research, the SMA‐proper seems to be primarily involved in controlled motor functions, such as motor initiation, motor triggering, and the temporal control of motor commands, which serve speech, vocalization, auditory, and movement (Bohland & Guenther, 2006; Hertrich et al., 2016; Lima et al., 2016). Findings from several studies on musical improvisation have implicated the SMA‐proper in the process of movement sequencing in spatial structures (Brown et al., 2006; de Manzano & Ullen, 2012; Limb & Braun, 2008), especially for externally‐triggered movements (Schwartze, Rothermich, & Kotz, 2012). Increased activity in the sensory‐motor areas (including PreG) may be associated with processing complex stimuli across multiple modalities in musical performance—which requires the integration of motor and auditory streams—consistent with previous work demonstrating that activation in the medial motor areas correlates with melodic sequence complexity (Bengtsson et al., 2007). In addition, long‐term practice for professional musicians may induce plastic changes within the cortical motor system and PFC, corresponding to decreased activation and increased functional segregation in these regions when performing complex motor sequences. In other words, musical training may facilitate higher‐level actions (e.g., high complexity) and creative performance (e.g., improvisation) without recruiting additional neuronal resources (Limb & Braun, 2008; Meister et al., 2005; Pinho et al., 2014).

Notably, musical creativity showed stronger engagement of left IFG compared to drawing creativity, but not literary creativity. One interpretation of this finding is that the left IFG supports the processing of musical syntax and semantics, such as the retrieval and selection of semantic information from long‐term memory (Koelsch, 2005; Moss et al., 2005; Pinho et al., 2016). Past work indicates that musical improvisation and verbal generation share similar neural substrates, including the left IFG (Brown et al., 2006). Moreover, a growing literature indicates that the left IFG is central to verbal divergent thinking, consistent with the notion that the generation of novel thoughts relies on overcoming dominant responses (Beaty et al., 2014; Benedek, Jauk, et al., 2014; Gonen‐Yaacovi et al., 2013). Collectively, musical creativity appears to be supported by domain‐specific activation of the left IFG and SMA‐proper, potentially reflecting the importance of musical‐semantic processing and long‐term motor specialization, respectively.

Regarding drawing creativity, our meta‐analysis revealed activation within the left fusiform gyrus, left precuneus, right parahippocampal gyrus, and right MFG. The involvement of fusiform gyrus in drawing creativity is perhaps not surprising, given that region's role in color/shape recognition and visual imagery (Ganis, Thompson, & Kosslyn, 2004; Ishai, Ungerleider, & Haxby, 2000)—processes central to performance on drawing tasks. A case study on visual creativity found that artists showed decreased activity in fusiform gyrus while drawing faces (Solso, 2001). Moreover, a study comparing experts and novices during a drawing task showed a smaller activated cluster in the fusiform gyrus in experts (Kowatari et al., 2009). Together, these findings indicate that the fusiform gyrus supports drawing creativity via basic visual perception and visual imagery, processes that are known to be shaped by expertise and training. Compared to musical creativity, drawing creativity recruited the precuneus and parahippocampal gyrus—regions of the default mode network, a network associated with spontaneous and self‐referential cognition, including episodic future thinking (Beaty et al., 2020; Schacter, Addis, & Buckner, 2007), autobiographical planning (Spreng, Gerlach, Turner, & Schacter, 2015), mind wandering (Christoff, Gordon, Smallwood, Smith, & Schooler, 2009), and self‐generated thought (Andrews‐Hanna, Smallwood, & Spreng, 2014). Beaty et al. (2014) proposed that the DN contributes to the generation of creative thought by extracting candidate ideas from long‐term memory; simultaneously, the control network, consisting of lateral PFC and IPL, evaluates and selects these candidate ideas to meet the constraints of task‐specific goals, such as originality and appropriateness (Beaty et al., 2016; Fan et al., 2014; Kowatari et al., 2009; Park et al., 2015). This view is supported by previous fMRI studies of drawing creativity (Ellamil et al., 2012) and literary creativity (Liu et al., 2015; Shah et al., 2013). For example, in drawing creativity, stronger activation in the medial temporal cortex (including hippocampus and parahippocampus) is related to the retrieval of novel ideas and construction of novel images during creative generation, whereas stronger activation in the precuneus may be implicated in information integration from the association cortex during creative evaluation (Ellamil et al., 2012). Moreover, a recent study reported that common neural activity within the parahippocampal gyrus during episodic retrieval, future imagination, and divergent thinking (Beaty, Thakral, Madore, Benedek, & Schacter, 2018), suggesting that common cognitive processes among drawing creativity and divergent thinking.

Interestingly, we found no significant difference in activation patterns between literary creativity and drawing creativity. However, considering that relatively less literature was available for literary creativity and drawing creativity, a more conservative and parsimonious interpretation is that the two artistic domains may recruit similar cognitive and neural mechanisms. A closer look at this small literature showed that subjects in the two domains were mostly nonexperts with minimal training; studies on music creativity, in contrast, were more likely to include experts in their samples. Another reason for a lack of difference between literary creativity and drawing creativity is that some studies only asked subjects to engage in mental imagery (Hahm et al., 2017; Howard‐Jones et al., 2005; Huang et al., 2013; Kowatari et al., 2009), not actual motor performance. This similar experimental design factor may thus partially explain the similarity in neural activity between drawing creativity and literary creativity. Moreover, compared to music, drawing and literary creativity may depend more on semantic and visual–spatial representations, not symbolic representations for music. As discussed above, drawing creativity and literary creativity are more associated with semantic processing, such as lingual gyrus, fusiform gyrus, and parahippocampus. This view is consistent with a prior study that contrasted writing and drawing, reporting similar activation associated with motor planning, language processing, and visuospatial mapping (Harrington, Farias, Davis, & Buonocore, 2007).

Beyond these common regions, literary creativity was especially associated with the left MTG and left LG, regions linked to language processing, semantic integration, and visual imagery. Prior research indicates that metaphor production is associated with activity in the peripheral temporal cortex (Beaty, Silvia, et al., 2017; Benedek, Beaty, et al., 2014; Benedek, Jauk, et al., 2014), which is crucial for sentence comprehension, prelexical perception, and semantic retrieval (Shah et al., 2013). This finding is in line with previous findings on verbal divergent thinking (Benedek et al., 2016; Fink et al., 2009; Wu et al., 2015), suggesting that occipitotemporal areas in verbal creativity might support novel idea generation via semantic information processing, mental imagery, and visual working memory (Chen et al., 2018; Chrysikou & Thompson‐Schill, 2011; Fink et al., 2009). Taken together, we found evidence that literary creativity and drawing creativity were associated with occipitotemporal areas involved in semantic and visual–spatial processing, with some regions potentially more specialized for literary creativity and drawing creativity, respectively. This pattern further suggests that domain‐general neural correlates seem associated with similar mental manipulation. Future research is needed to explore the similarities in neural activation and basic cognitive operations between different creative forms.

### 4.3 Limitations and future directions

Several well‐known limitations have been discussed in image‐based meta‐analyses, such as publication bias or file‐drawer effect (Lipsey & Wilson, 1993), heterogeneity of experimental conditions and contrasts, and variation in data‐analysis procedures (Müller et al., 2018). In the context of creativity research, one common criticism of meta‐analyses is the variance in the operational definition of creativity, which could impede the identification of neural regions involved in artistic creativity for each domain. For example, although the central concept of drawing creativity concerns the production of novel and esthetically‐pleasing visual‐forms, experimental procedures used to measure creativity are different, with some studies focusing on imagining how to design novel products (Hahm et al., 2017; Huang et al., 2013; Kowatari, et al., 2009) and other studies focusing on idea generation on the spot (Ellamil et al., 2012; Fan et al., 2014; Saggar et al., 2015; Saggar et al., 2017). Moreover, different control conditions have been used to contrast against creative conditions; for example, in musical creativity, rest, random button presses, familiar patterns, and memory retrieval were used as control conditions in different studies. In sum, measurement variation in creativity studies would result in divergent findings, making it difficult to compare and integrate findings within or across different domains. In light of these issues, previous reviews suggested that research should carefully consider the psychometric properties of creative cognition and revise creative measures, making them more reliable and valid (Arden et al., 2010). Besides, another limitation of neuroimaging meta‐analyses is that most coordinate‐based algorithms may be insensitive to nonsignificant results, leading to publication bias due to various data‐analysis approaches as well as flexibility in inference and thresholding for a significant result (Carp, 2012). Here, we mainly address two potential limitations based on the present results in the context of the status quo of creativity neuroscience.

First, the shared neuronal activation across the three artistic forms was only based on the overlay in the spatial pattern. It is important to note that these spatial co‐activation regions do not necessarily indicate similar functional activation patterns and equivalent cognitive functions (Hawes, Sokolowski, Ononye, & Ansari, 2019). Although we discussed their commonality and cognitive role in creative thinking, whether or not the same functionally meaningful brain regions overlap in the same participants across different artistic modalities remains an open question. Therefore, one important and promising future direction is to explore whether similar activation patterns exist within overlapping regions while artistic engage in their respective domain. Although challenging, given different levels of expertise required for each creative domain, it would be interesting to compare multiple artists in the same study with varying levels of expertise across music, drawing, and literary creativity. In this way, one could decode patterns of activation within overlapping regions across multiple creative domains, revealing domain‐general and domain‐specific brain regions along with their corresponding cognitive correlates.

Second, creative thinking is a complex and dynamic process that requires multiple cognitive processes, including higher‐order cognition (divergent and convergent thinking), fundamental cognitive mechanisms (e.g., attention, working memory, and cognitive control), and the interplay between these cognitive processes (Mekern, Hommel, & Sjoerds, 2019). Generally, creative thinking is viewed as a dynamic process of idea generation and idea evaluation, dependent upon process‐related neural networks and their interaction. Although the present meta‐analysis mostly focused on the phase of idea generation, it remains difficult to define clear‐cut boundaries to separate idea generation from idea evaluation during creative thinking and artistic performance. Likewise, a significant challenge lies in how to separate idea generation and evaluation in an ecologically valid experimental procedure, and whether parsing creative performance into discrete stages artificially distorts the naturalistic process of artistic creation. Although previous research on the twofold model has provided many insights (Ellamil et al., 2012), the two stages can be arbitrarily imposed by the experimental design, thus potentially affecting ecological validity and highlighting the difficulty of capturing the creative brain (Sonkusare, Breakspear, & Guo, 2019). Cutting‐edge statistical analyses, such as Multi‐Voxel Pattern Analysis (MVPA) and hidden semi‐Markov models (HSMM; Anderson, Pyke, & Fincham, 2016), may offer a promising approach to decoding cognitive processes in a more naturalistic experimental context. In sum, combining naturalistic approaches with the twofold framework may help to clarify the domain‐general and domain‐specific mechanisms of creativity, providing greater clarity into the complex neural underpinnings of creative cognition and artistic performance.

## 5 CONCLUSION

Whether creativity relies on domain‐general or domain‐specific cognitive processes remains an open question. Many behavioral studies have explored potential domain‐general and domain‐specific mechanisms by investigating associations between distinct creative tasks. Recent neuroimaging research has demonstrated consistent prefrontal activation during verbal, visuospatial, and musical creativity, but a systematic framework to interpret these findings has so far been lacking. Such a neuroscience framework can enrich current theories of creativity and motivate a promising direction to contextualize future work on the neurocognitive basis of creative thinking. The present study aimed to uncover the domain‐mechanisms of artistic creativity by performing an ALE meta‐analysis on brain regions associated with three forms of creative performance. Consistent with our hypotheses, results revealed that the three creative domains all recruited the pre‐SMA, left DLPFC, and right IFG, suggesting that these regions support domain‐general processes during artistic creation. We also found that some regions were more specialized for one type of artistic creativity, such as MTG and right lingual gyrus for literary creativity, suggesting that domain‐specific processes are also important for artistic creativity. Taken together, these findings provide a path forward for future investigations of artistic creativity, emphasizing the need to dissociate domain‐general vs. domain‐specific neural systems underlying creative performance.

## CONFLICT OF INTERESTS

The authors declare that they have no conflict of interest.

## Data Availability

The data that support the findings of this study are available from the corresponding author upon reasonable request.
